# *Candidozyma auris* Reported in Scotland: a Call for Vigilance Amid Global Rise

**DOI:** 10.1007/s11046-026-01051-y

**Published:** 2026-01-20

**Authors:** M. H. Howe, K. L. Bartie, W. G. Mackay, T. Inkster, S. Cairns, R. Kean, A. M. Bal, G. Ramage

**Affiliations:** 1 Safeguarding Health Through Infection Prevention (SHIP) Group, School of Health and Life Sciences, Glasgow Caledonian University, Glasgow, UK; 2 Antimicrobial Resistance and Healthcare Associated Infection (ARHAI) Scotland, Glasgow, UK; 3 Institute of Healthcare Policy and Practice, School of Health, Nursing, and Midwifery, University of the West of Scotland, Paisley, UK; 4 Department of Microbiology, NHS Greater Glasgow & Clyde, Glasgow, UK

**Keywords:** *Candidozyma auris*, *Candida auris*, Surveillance, Emerging fungal pathogen

## Abstract

*Candidozyma auris* (formerly *Candida auris*) is an emerging pathogenic yeast associated with healthcare outbreaks worldwide. Despite increasing reports across Europe, no published data have previously described cases in Scotland. Here, we report the first detections of *C. auris* in Scotland, as submitted to ARHAI Scotland. Eight cases (seven colonisations, one infection) were identified to date across four NHS Scotland boards, all linked to repatriation or recent hospitalisation abroad. To contextualise these findings, we reviewed publicly available literature and surveillance data for Western and Northern Europe, identifying considerable variation in case numbers and highlighting Scotland’s position among countries with the lowest reported cases. All Scottish cases were imported, underscoring the importance of targeted screening of patients with international healthcare exposure. These findings inform preparedness planning and support recommendations for strengthened surveillance to prevent onward transmission.

## Short Communication

The yeast pathogen *Candidozyma auris* (formerly *Candida auris*) is increasingly recognised as a significant cause of healthcare-associated infections worldwide since its first isolation in 2009 [1]. Its ability to form biofilms, which enhances resistance and promotes persistence, presents a major challenge in healthcare settings [2]. *C. auris* is a resilient pathogen, evidenced by recent work where dry surface *C. auris* biofilms became increasingly resistant to the 1000 ppm recommended levels of sodium hypochlorite for disinfection [3]. The WHO categorised *C. auris* as a critical priority pathogen in 2022, highlighting the need to strengthen the global response to fungal infections and resistance. Since then, leading experts from across the globe have collaborated to publish new guidelines for the management of *Candida* infections [4].

Worldwide, 61 countries had reported cases and outbreaks of *C. auris* by the end of 2023 [5]. Yearly surveys done by the European Centre for Disease Prevention and Control (ECDC) revealed that cases are rapidly increasing in countries of the European Union (EU)/European Economic Area (EEA) [6]. In the EU/EEA, the first case was reported in 2014, with only three cases reported in 2015 but since then the incidence has rapidly increased from 804 cases in 2022 across 14 countries, to 1346 in 2023 across 18 countries [6]. In England, 637 cases of *C. auris* were documented collectively between January 2013 and the end of December 2024, with 178 cases identified in 2024 alone according to the United Kingdom Health Security Agency’s (UKHSA) Health Protection Report (Volume 19, Issue 3) published this year [7]. The UKHSA *C. auris* guidance for acute healthcare settings has been updated in response to this rise of cases [8].

Currently, no published literature has described *C. auris* cases or outbreaks in Scotland. Under the minimum alert organism list within the National Infection Prevention and Control Manual (NIPCM), NHS Scotland boards are encouraged to report *C. auris* cases to Antimicrobial Resistance and Healthcare Associated Infection (ARHAI) Scotland [9]. Here, we review the reported incidents of *C. auris* submitted to ARHAI Scotland until October 2025 to establish the national baseline, providing a reference point for future surveillance and comparison. To contextualise these findings, we compared case numbers in Western and Northern European countries, chosen with consideration of the United Nations geoscheme and their broadly similar environmental and healthcare contexts [10]. A targeted PubMed search was conducted using the terms “Candida auris” and “Candidozyma auris” in combination with individual country names. Reference lists were reviewed to identify additional relevant publications and publicly available data were included from national surveillance programmes and other grey literature sources not typically indexed in PubMed.

To date, eight *C. auris* cases (seven colonisations and one infection) have been reported to ARHAI Scotland across four NHS Scotland boards using the outbreak reporting tool (ORT). No cases were reported before 2023, four cases were reported in 2023, one was reported in 2024 and three cases in 2025. All cases had a history of repatriation from or recent admission to healthcare facilities outside Scotland of which one was transferred from England, four from Greece, one from United States of America, one from United Arab Emirates, and one from South Africa. One case was detected from a *C. auris* screen undertaken because of a positive carbapenemase producing organism (CPO) screen, following existing recommendations. Seven cases were detected through screening samples undertaken on repatriation or history of recent hospitalisation overseas, highlighting the role of targeted surveillance in early detection from at risk populations with recent international travel. One patient had a bloodstream infection. Four isolates were assigned to Clade I (three from Greece and one from the United Arab Emirates), whereas the isolate from the United States of America was assigned to Clade IV. Three isolates were not retained due to resource constraints and lack of clear guidance; therefore, strain specific information is unavailable.

**Fig. 1:**
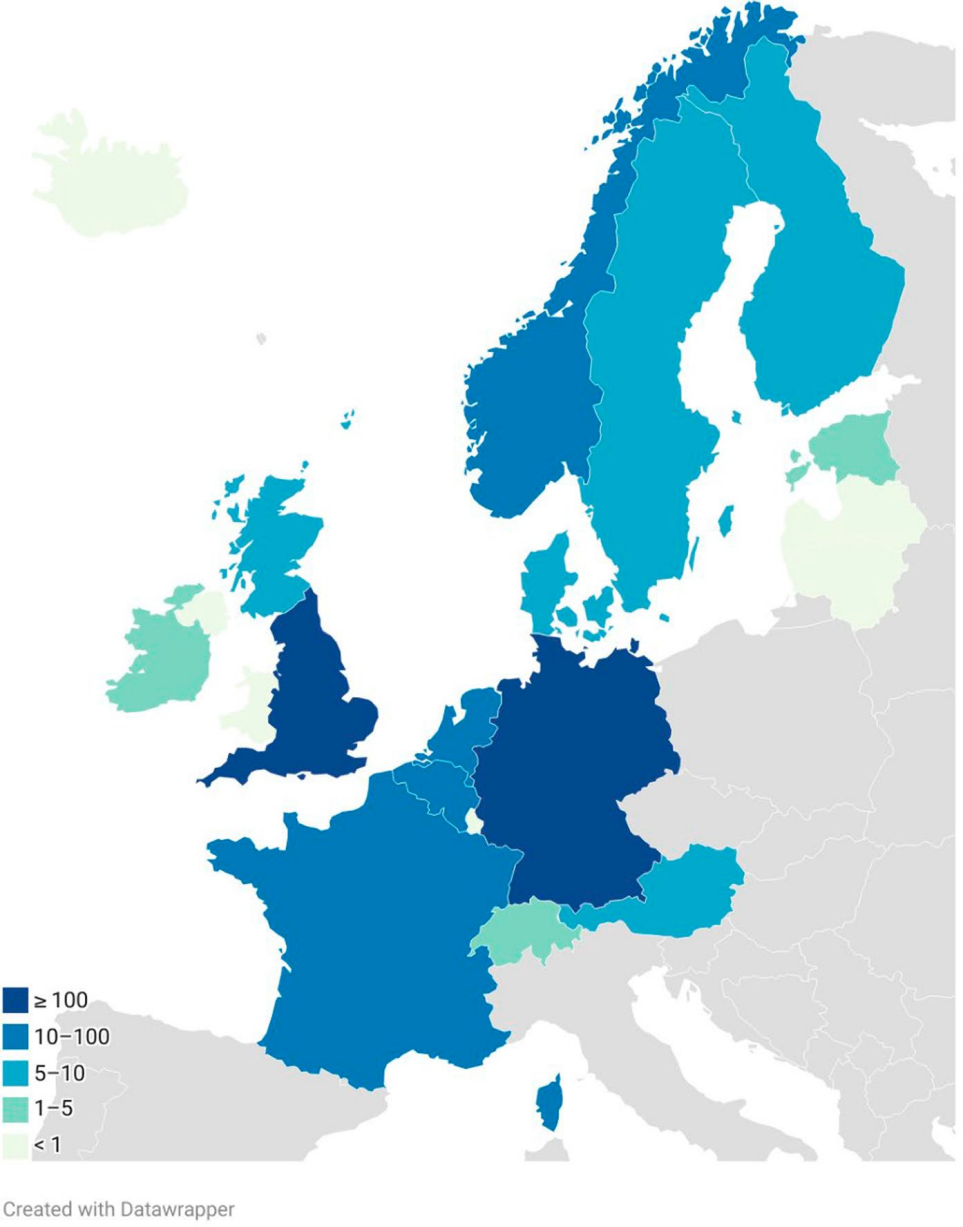
Geographic distribution map of minimum reported *Candidozyma auris* cases in Western and Northern Europe. Colours indicate case ranges: light green = no cases, green = 1–5 cases, light blue = 5–10 cases, blue = 10–100 cases, dark blue =  ≥ 100 cases. Grey = countries outside Western and Northern Europe. Data compiled from published literature, government sources, and publicly available surveillance reports (see Table 1)

Confirmed cases, based on published literature, government sources and other publicly available data available, show a varied distribution across Western and Northern Europe (Fig. 1 and Table 1). These numbers represent the minimum cases identified, as case reporting is not standardised and delays in publication are common. Twenty-two countries were included in our analysis of which eight reported no cases and eight reported fewer than ten cases. Scotland’s eight cases place it among countries with the lowest reported numbers, alongside Austria (n = 7), Sweden (n = 6), Denmark (n = 5), and Finland (n = 5). These countries share relatively colder climates, which may contribute to reduced environmental persistence of *C. auris* compared to warmer regions. Although current case numbers are low in colder climates, evidence indicates that temperature influences fungal ecology and global warming may enable thermotolerant pathogenic fungi, including *C. auris*, to adapt and spread into regions that were previously unsuitable due to lower temperatures [11–13]. Nevertheless, the presence of cases despite cooler conditions indicates that climate alone does not eliminate risk, particularly in healthcare settings where transmission can occur. Moreover, these countries also have relatively small populations, which may reduce the scale of population movement and associated opportunities for the introduction and onward transmission of *C. auris*. The highest numbers were observed in England (n = 637) followed by Germany (n = 145) and France (n = 55). Countries with warmer climates (e.g., Spain, Greece, Italy, Romania) are reported by the latest ECDC survey including EU/EEA countries as having the highest cumulative case numbers, suggesting that temperature may influence persistence of *C. auris* [6].

**Table 1:** Minimum reported *Candidozyma auris* case numbers by mainland country in Western and Northern Europe

Country	Minimum case number	Date last reported/updated	Reference(s)
Austria*	7	December 2023	[6, 17]
Belgium	21	September 2025	[6, 18, 19]
Denmark	5	December 2023	[6, 20]
England (UK)	637	December 2024	[7]
Estonia*	1	December 2023	[6]
Finland*	5	December 2023	[6]
France	55	December 2023	[6, 21, 22]
Germany	145	September 2025	[6, 23, 24]
Netherlands*	26	June 2025	[6, 25]
Norway*	19	September 2025	[6, 26]
Republic of Ireland*	4	June 2023	[6, 20]
Scotland (UK)*	8	October 2025	This study
Sweden*	6	June 2024	[6, 27]
Switzerland	3	September 2020	[28, 29]

^*^ = all imported cases. No reports were found for the following countries: Iceland, Latvia, Liechtenstein, Lithuania, Luxembourg, Monaco, Northern Ireland (UK), Wales (UK)

Interestingly, out of the 14 countries that have reported one or more *C. auris* cases, eight had imported cases only (Table 1). This suggests that, for many countries, *C. auris* does not have established local transmission yet as noted in Scotland. Cross-border patient movement remains the primary route of introduction which reinforces the importance of screening individuals with recent international hospitalisation to prevent onward transmission. A similar early pattern was observed in England, where sporadic imports were initially reported, and outbreaks have since been documented leading to invasive cases [14]. Once established, *C. auris* can be highly transmissible in healthcare settings, spreading through direct and indirect contact, with reusable equipment of particular concern [15]. A French study demonstrated that patient-to-patient transmission of *C. auris* occurred between 41 and 61 days after initial exposure, despite multiple negative screening results leading up to that point [16]. This finding supports the need for enhanced screening protocols to limit transmission and highlights the importance of retaining isolates for future investigation.

Gaps in national reporting systems suggest the *C. auris* cases presented here may be underestimated. A recent report from the ECDC, based on survey responses from participating countries, revealed that only 17 out of the 36 EU/EEA countries have a national surveillance system in place [6]. Of those, only nine countries require mandatory reporting which includes the following Western and Northern European countries: Belgium, Denmark, Germany, Iceland, Republic of Ireland, Luxembourg, and Norway [6]. Since April 2025, reporting of *C. auris* in England is mandatory [30]. However, reporting is not mandatory in Scotland, although this is strongly encouraged, which may contribute to underreporting of cases. Moreover, diagnostic limitations could underestimate the prevalence of *C. auris*. Accurate identification of *C. auris* in the laboratory setting remains challenging, as it is frequently misidentified due to its phenotypic similarity to other closely related species such as *C. haemuli, C. duobushaemuli* or *Clavispora lusitaniae*.[31–33] Although a range of diagnostic tools are available internationally, including molecular methods such as whole genome sequencing [34], access to these technologies within Scottish health boards remains limited. Current practice for *C. auris* identification within NHS Scotland typically involves culture-based methods, followed by MALDI-TOF MS using an updated database that includes *C. auris* after which results should be confirmed by the UKHSA Mycology Reference Laboratory, Bristol. Constraints related to funding, infrastructure, and specialist expertise may limit the routine use of advanced diagnostics, potentially contributing to missed or delayed detection and lack of retention of isolates for further investigation.

This is the first report of detection of *C. auris* cases in Scotland. In 2023, ARHAI Scotland issued a briefing note to all NHS Scotland health boards recommending screening of repatriated patients and newly identified CPO-positive cases, which has led to the detection of eight cases to date. The identification of *C. auris* following a positive CPO result highlights the value of using colonisation with other multi-drug resistant organisms as a trigger for screening, as this case may have been missed without Scotland’s policy to screen CPO-positive patients. Compared to some European countries, including England, the number of cases in Scotland remains low. This may be influenced by Scotland’s colder climate, and when compared to other colder regions the figures appear broadly similar. However, underreporting of cases is possible due to limited diagnostic capacity, challenges in accurate identification and the absence of mandatory surveillance for *C. auris* for Scotland. Scottish Government are currently considering the addition of *C. auris* to Schedule 2 of the Public Health etc. (Scotland) Act 2008 as a notifiable organism. This will be crucial to support comprehensive and timely identification. National discussions are ongoing to revise existing guidance, and NHS Scotland Boards and IPC teams are encouraged to assess their preparedness. Strengthening surveillance and screening measures will be critical to limiting transmission and preventing progression beyond the sporadic imported cases currently observed in Scotland, particularly given the organism’s ability to persist in healthcare environments.

## Data Availability

No datasets were generated or analysed during the current study.
